# Climate Change and Healthy Aging: What Are the Existing Data in Aging Studies?

**DOI:** 10.1093/geroni/igaf008

**Published:** 2025-02-12

**Authors:** Yu-Tzu Wu, Matthew Prina, Paola Zaninotto

**Affiliations:** Population Health Sciences Institute, Faculty of Medical Sciences, Newcastle University, Newcastle upon Tyne, UK; Population Health Sciences Institute, Faculty of Medical Sciences, Newcastle University, Newcastle upon Tyne, UK; Department of Epidemiology and Public Health, University College London, London, UK

**Keywords:** Behaviors, Climate action engagement, Climate-related worry, Knowledge, Preparedness

**Translational Significance:** Climate change poses a significant threat to older populations, yet there is insufficient data on its impact specific to them. Despite this, current aging studies lack climate change measures concerning older adults. This work highlights the need for climate-focused questionnaires in aging studies, enabling a more comprehensive understanding of older adults’ responses to climate change, their preparedness for climate-related extreme events, climate change perceptions, and their potential involvement in pro-environmental activities. Integrating climate data into aging studies and linking it to climate event exposure can support robust research, guiding evidence-based policies and practices to protect vulnerable older populations.

Older people, who often experience declines in health and functioning, are particularly vulnerable to the effects of climate change. For example, extreme heat can exacerbate existing health conditions like cardiovascular and respiratory diseases, whereas increased frequency of severe weather events, such as floods or hurricanes, can lead to physical injury, displacement, and heightened stress levels in older populations (1). To address the complex challenges posed by climate change and its impact on healthy aging, here defined as the process of developing and maintaining the ability to function well in older age (2), there is an urgent need to develop robust research and inform evidence-based policies and practice.

In our systematic mapping, we aimed to identify aging studies that collected measures related to climate change, including data on exposure through linkage or surveys, as well as climate change questionnaires. Specifically, we focused on 2 types of measures: (1) climate exposures (eg, temperature, heat, precipitation) and (2) questionnaires evaluating knowledge, concerns, attitudes, perceptions, and behaviors regarding climate change. This approach was intended to provide a comprehensive overview of the data available in this field. Our systematic mapping approach targeted studies involving older adults aged 50 and above, identifying 153 studies through 3 major aging research consortiums selected for their distinct strengths. The Integrative Analysis of Longitudinal Studies of Ageing and Dementia (IALSA; *N* = 119) (3) is the largest network of individual studies on aging, Ageing Trajectories of Health: Longitudinal Opportunities and Synergies (ATHLOS; *N* = 18) (4) focuses specifically on healthy aging, and the Gateway to Global Aging Data (g2aging.org; *N* = 16) (5) is the most widely used network of aging studies. Leveraging these networks enabled us to include a diverse collection of studies with strong geographical representation of aging populations worldwide.

Our process involved 2 key steps. First, we reviewed study websites, documents, and profiles to identify climate change-related measures documented in publicly accessible sources. Second, between April and May 2024, we conducted a survey targeting the consortium aging studies. We sent an email to the principal investigators and study teams, inquiring whether they had collected data on climate exposures, administered relevant questionnaires, or had plans to incorporate such measures in the future.

We reviewed the 154 studies in the three consortiums and selected 78 relevant studies based on the following criteria: (1) age of the respondents 50+; (2) collected or linked data about climate change; and (3) were ongoing. We contacted each of the 78 relevant ongoing studies of people aged 50 or above. Of these, 41 (52%) responded to our survey and are included in this work. We compared the responding and nonresponding studies based on country of origin, cohort starting year, and sample size, and we found no statistically significant or systematic differences between these 2 groups, suggesting that nonresponse bias is unlikely to have impacted our findings. Out of the 41 we identified 10 studies with included climate exposures such as temperature (eg, heat, cold), rainfall (eg, humidity), and sunlight (Table 1). Most of the studies were from high-income countries, including Canada (*N* = 4), Australia (*N* = 2), Ireland (*N* = 1), the Netherlands (*N* = 1), the United States (*N* = 1), and Costa Rica (*N* = 1). These exposure measures were derived from meteorological data and linked to the cohort studies based on participants’ residential locations. Two studies, the German Socio-Economic Panel Study (Germany) and Hunter Community Study (Australia), included questionnaires developed nearly 10 years ago on sustainability behaviors and Solastalgia, which focuses on distress caused by environmental changes (6). However, these questionnaires did not directly address climate change issues. Lastly, the English Longitudinal Study of Ageing, included in its latest data collection (still underway at the time of this work) 6 items on negative climate change beliefs, environmental worldview, and environmental lifestyle. These items were administered as part of Understanding Society, a survey covering individuals of all ages in the general population, and were not specifically developed for use in aging studies.

**Table 1.** Aging Studies Including Climate Exposures (*N* = 10) and Questionnaires (*N* = 2)

Study (Acronym)	Country	Content
Climate exposure		
Alberta’s Tomorrow Project (ATP)	Canada	Meteorological exposures from the Canadian Urban Environmental Health Research Consortium (CANUE)
British Columbia Generations Project (BCGP)	Canada	Meteorological exposures from the Canadian Urban Environmental Health Research Consortium (CANUE)
Canadian Partnership for Tomorrow’s Health (CanPath)	Canada	Meteorological exposures from the Canadian Urban Environmental Health Research Consortium (CANUE)
Canadian Longitudinal Study on Ageing (CLSA)	Canada	Meteorological exposures from the Canadian Urban Environmental Health Research Consortium (CANUE)
Sydney Memory and Aging Study (SydneyMAS)	Australia	Heat, rain, humidity, sunlight, temperature, wind speed
International Mind, Activities and Urban Places study (iMap)	Australia	Temperature, relative humidity and precipitation
Costa Rican Longevity and Healthy Aging Study (CRELES)	Costa Rica	Heat/temperature
The Irish Longitudinal Study on Ageing (TILDA)	Ireland	Seasonal and meteorological conditions: temperature, rainfall, sunshine
The English Longitudinal Study on Ageing (ELSA)	England	Climate change beliefs, environmental worldview, and environmental lifestyle
Longitudinal Aging Study Amsterdam (LASA)	The Netherlands	Temperature, urban heat island
Nurses’ Health Study (NHS)	United States	Temperature
Questionnaires		
German Socio-Economic Panel Study (SOEP)	Germany	General Ecological Behavior Scale, Sustainable Consumption Behavior
Hunter Community Study (Hunter CS)	Australia	Solastalgia

Through our systematic mapping, we found that only a limited number of existing aging studies included questionnaires specifically addressing climate change. To advance research on the intersection of climate change and healthy aging, studies should incorporate climate-related measures into their data collection that are relevant to older age. Developing tailored questionnaires for older adults is crucial for aging research. Recent surveys (7,8) of the general population have highlighted the importance of collecting data on climate change-related concerns, attitudes, behaviors, beliefs, knowledge, and perceptions; however, these domains have not been specifically the focus in older adults.

Furthermore, the impact of climate change on the human rights of older people has been acknowledged by the United Nations and ageism is recognized as a key issue in addition to life, health, and safety (9). Bias against older people being “frail, sick and dependent” may lead to marginalization and exclusion in policy and practice (10). These stereotypes often extend to climate change contexts, where older people are unfairly viewed as passive, disengaged, or even disproportionately responsible for the current environmental crisis (9,10). However, contrary to these misconceptions, older adults actually represent a significant reserve of human capital (11) that is vital for addressing and preventing climate change (12). With increased healthy longevity and fewer familial and professional obligations, older individuals have the potential for sustained and meaningful pro-environmental engagement (13). Therefore, it is essential to adapt or develop questionnaires that accurately capture the unique perspectives of this age group.

This systematic mapping highlighted a significant lack of focused data on older adults in the context of climate change. Despite widespread ageist stereotypes, there is evidence that older adults have considerable potential for meaningful climate action which, when combined with theoretical insights, underscores the need for targeted approaches. Therefore, we recommend prioritizing the following areas in surveys specifically designed for older populations:

(1) Climate worry: Older adults may experience increased worry and anxiety due to the impacts of climate change on the environment, landscapes, wildlife, and human communities (6). Climate worry should be assessed as part of a survey for older people to discuss their experience.
(2) Attitude and behaviors: Older adults are often stereotyped as passive, disinterested, or withdrawn, and are sometimes unfairly accused of disproportionately contributing to climate change (9,10). Questions on attitudes and behaviors can provide empirical data to challenge these biases and highlight the heterogeneity of this age group.
(3) Preparedness for extreme events: Older people are particularly vulnerable to the effects of climate change such as extreme heat, floods, and hurricanes, which can adversely impact both physical and mental health (1). Questions on preparedness can help evaluate how older people prepare themselves to mitigate the climate impacts on health.
(4) Climate action involvement: Retirement and healthy longevity offer older adults opportunities to engage in pro-environmental activities (13). Questions in this area are crucial for exploring the current involvement of older adults in climate action and their potential to contribute further to environmental causes.

These areas are crucial for enhancing empirical data on climate change and healthy aging. When paired with respondents’ geographic location—a factor that many survey studies could potentially leverage—this could enable linkage to climate data (eg, extreme weather events, heat, etc.) and ultimately allows survey studies to more accurately assess individuals’ risk of climate change impacts. Furthermore, including climate-related questionnaires in aging studies will deepen our understanding of climate impacts on the physical, mental, and social health of diverse older populations, including those with chronic conditions and disabilities. This will also support evidence-based policy-making in public and environmental health.

## Data Availability

Data and material in this study are available upon request to the corresponding author. The study was not preregistered.
